# Prevalence of schistosome infection in a region of Madagascar regularly undergoing mass drug administration: a cross-sectional study

**DOI:** 10.1080/20477724.2026.2616620

**Published:** 2026-02-02

**Authors:** Ralf Krumkamp, Aaron Remkes, Jacques Hainasoa, Tahinamandranto Rasamoelina, A. Ravo Razafindrakoto, N. Mathieu Razafindralava, Jana C. Hey, Doris Winter, Natalie Fischer, Leonard Gunga, Philippe Martel, Nicolas Jouanard, Zo Andrianarinirina, Pia Rausche, Jean-Marc Kutz, Cheick O. Doumbia, Raphaël Rakotozandrindrainy, Jürgen May, Valentina Marchese, Rivo A. Rakotoarivelo, RESAMP Consortium, Daniela Fusco

**Affiliations:** a Department of Infectious Diseases Epidemiology, Bernhard Nocht Institute for Tropical Medicine (BNITM), Hamburg, Germany; b German Center for Infection Research (DZIF), Hamburg-Borstel-Lübeck-Riems, Germany; c Department of Infectious Diseases, University of Fianarantsoa Andrainjato, Fianarantsoa, Madagascar; d Centre d’Infectiologie Charles Mérieux (CICM), University of Antananarivo, Antananarivo, Madagascar; e APDRA Pisciculture Paysanne, Massy, France; f Department of epidemiology, University Clinical Research Center, University of Sciences, Techniques and Technologies of Bamako, Bamako, Mali; g Department of Microbiology and Parasitology, University Antananarivo, Antananarivo, Madagascar

**Keywords:** Schistosomiasis, MDA, public health

## Abstract

Schistosomiasis is a parasitic disease primarily controlled by Praziquantel-based Mass Drug Administration (MDA) targeting school-aged children. This study aimed to generate a high-resolution schistosomiasis prevalence map in a region with regular MDA in Madagascar, identify at-risk groups, assess population knowledge, and explore risk factors to support alignment with WHO guidelines. Conducted between July and October 2022 in the District of Vatomandry, Madagascar, this cross-sectional study included participants aged five and older. Point-of-Care Circulating Cathodic Antigen testing determined infection prevalence. A choropleth map was generated to show the geographical distribution of schistosome infection across local communities. Individual risk factors were assessed using hierarchical Poisson regression. The study included 1,215 households (5,200 participants) from 42 communities revealing a high overall prevalence of 59%. Adjusted prevalence ratios (PR) indicated higher infection risks for farmers (PR = 1.17, 95% CI: 1.09–1.25) and older age groups (≥38 years: PR = 1.34, 95% CI: 1.16–1.55; against ≤15 years), while participants with higher education or knowledge about schistosome transmission showed a reduced risk (PR = 0.88, 95% CI: 0.78–0.99). The results show high schistosome prevalence in adults, emphasizing the need to adapt strategies in endemic countries to meet the WHO’s 2030 goal of eliminating schistosomiasis as public health problem.

## Introduction

Schistosomiasis is a parasitic disease associated with and perpetuating poverty [1]. The disease is vector-borne, transmitted by freshwater snails, and spread through contaminated waters [2,3]. The transmission cycle is highly perpetuated in regions with an agricultural-based economy [4,5]. Despite the World Health Organization’s (WHO) recommendations to target public health interventions against schistosomiasis to all groups at-risk (e.g. high-exposure occupations such as fishermen, occupational washers, irrigation farmers, or women doing domestic chores in contaminated waters) in countries with a prevalence greater than 10%, Mass Drug Administration (MDA) with Praziquantel (PZQ) – the main public health intervention against the disease – still predominantly focuses on school age children (SAC) [6].

MDAs have proven to be relatively cost-effective and have significantly contributed to the successful elimination of disease transmission and/or the elimination of different Neglected Tropical Diseases (NTDs) as a public health concern [7]. However, in many cases, MDAs have failed to achieve success in disease control and transmission interruption. In the case of schistosomiasis, it has been shown that where prevalence is below 10%, achieving 75% MDA coverage at annual intervals is considered sufficient for elimination efforts [8]. In communities where prevalence exceeds 10%, MDAs targeting SAC, even with high coverage, prove to be insufficient for eliminating the disease or adequately controlling transmission as numerous at-risk groups, such as adults, are not reached at all [6]. The recently released WHO schistosomiasis guidelines recommend MDAs in endemic communities with a prevalence surpassing 10% for all individuals at risk aged two years and older [9]. Additionally, the WHO recommends that MDAs should be complemented by a comprehensive One Health approach, including improvements in water, sanitation, and hygiene (WASH), implementation of environmental and ecological measures, snail control, and targeted health education for at-risk populations [10–13]. Nevertheless, many endemic countries, most of which are situated in sub-Saharan Africa (SSA), continue to lag behind schedule in updating their national guidelines [14,15]. Shifting the intervention from SAC to all at-risk groups requires significant commitment from local authorities, who must reconceptualize longstanding programs with both financial and structural implications.

A notable example is Madagascar, which, despite achieving high MDA coverage, is still struggling to align with the WHO’s objective of eliminating schistosomiasis as a public health problem by 2030 [14,16]. Recent studies indicate that schistosomiasis prevalence in the population remains persistently high [17,18]. Despite the long history of MDAs in the country, barriers to implementing the most recent WHO guidelines persist [14]. Moreover, higher-resolution estimates of prevalence – such as at the community, household, or specific at-risk group level – have not yet been well established. As per current practice, monitoring and evaluation programs are conducted at the district level in Madagascar [16], typically using sentinel site surveys among SAC to assess whether prevalence exceeds the 10% WHO-recommended threshold, which in turn guides the frequency and coverage of MDA strategies [14]. This approach may mask within-district heterogeneity, underscoring the need for more granular data to better tailor interventions.

The objective of this study was to determine the prevalence of schistosome infection in the Vatomandry District of Madagascar, an *S. mansoni* endemic area [19] with systematic, high-coverage MDA interventions among SAC, particularly where rice cultivation is the predominant occupation following WHO recommendations [13]. The goal was to advocate for the adaptation of national guidelines and the development of effective strategies to eliminate schistosomiasis as a public health problem by 2030.

## Methods

### Study setting

**Figure 1.**
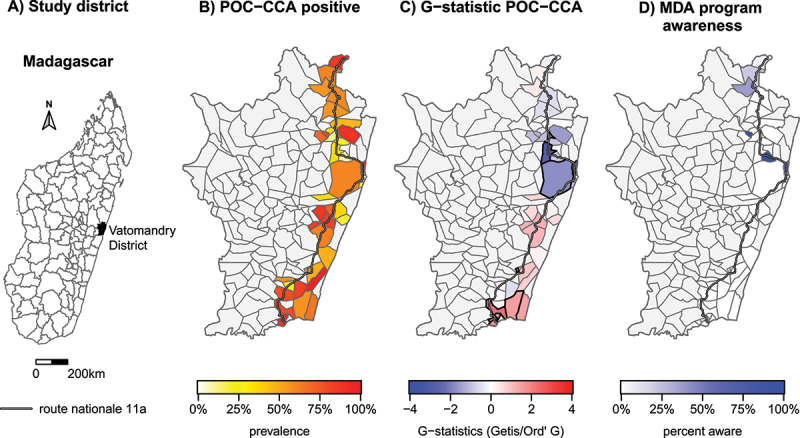
Maps displaying (A) the District of Vatomandry in Madagascar, (B) the percentage of POC-CCA positive participants, (C) local spatial G-statistics indicating hot and cold spots for schistosomiasis (Fokontany outlined by a bold border show a G-statistics p-value <0.1), and (D) the proportion of participants aware of MDA programs in their villages. Maps B to D cover the entire District of Vatomandry with all its Fokontany, with those not included in the study shaded grey. The national road 11a is also depicted on the maps.

This cross-sectional study was conducted from the 21st of July to the 17th of October 2022 in the District of Vatomandry, in the Region of Antsinanana on the east coast of Madagascar (Figure 1(A)). A total of 42 out of 208 Fokontany, the smallest administrative subdivisions at the commune level in Madagascar, were selected in the district based on accessibility by motorbike and their proximity to the Vatomandry hospital (*Centre Hospitalier de Référence du District de Vatomandry*), within a two-hour drive. The close proximity facilitated the daily transportation of those biological specimens for processing and storage, although these specimens were not included in the present study. Based on the last census (2018), these Fokontany represent a total population of 53,799 out of 170,996 for the whole district, with a median Fokontany population of 1,135 (IQR = 744–1,891). The last MDA in the region prior to the study was conducted between 2020 and 2021 [14,16]. Participants were considered to be eligible exclusively if living in the selected Fokontany.

### Participants recruitment

The sample size was estimated for a binary outcome using two-stage cluster sampling to calculate the prevalence of schistosome infection. Based on similar studies conducted in other districts of Madagascar [17,18] we assumed a prevalence of 60%. This proportion was estimated with a precision of 5% at a 95% confidence level (CI), assuming an intra-cluster correlation at Fokontany-level of 0.02. In total, 3,468 individuals in 29 clusters were deemed necessary for the study. The target was to sample 10% of each Fokontany’s population, adjusted based on the baseline population size of each Fokontany (according to the 2018 census), with approximately 120 individuals selected per Fokontany. During the course of the study, we had the opportunity to include additional Fokontany. However, we maintained the relative number of individuals to be sampled per cluster. Ultimately, 42 Fokontany were included in the study, resulting in a sample size of 5,200 individuals.

Prior to each site sampling, primary healthcare centers (PHC) and the chiefs of each Fokontany were informed about the study and asked for permission. The inclusion criteria for the study were age ≥5 years, informed consent given, no current fever or COVID-19 infection, and no COVID-19 vaccination within the past 2 weeks. Since the study was conducted outside healthcare facilities, certain conditions were excluded to avoid potential adverse events. These included a history of epilepsy or convulsive seizures (which could suggest cysticercosis), pregnancy (due to limited data on PZQ use in the first trimester), and other health issues like COVID-19 or fever. These conditions were excluded to prevent worsening or masking potential side effects of PZQ [20] that was offered to participants tested positive for schistosomiasis. For underaged participants, assent was obtained in addition to written informed consent from a legal guardian, who was also required to be present during the survey.

A randomization and sampling procedure was designed to minimize selection bias and ensure inclusivity across socio-economic groups and daily schedules of participants. Within each Fokontany, fixed landmarks like community halls, churches, or schools were randomly selected as starting points from where a compass application [21] determined a random direction, setting a path for selecting every second household on the left and right. These households were informed a day prior to sampling to ensure availability of all household members. On the sampling day, a randomizer application [22] selected up to five participants per household from those present, ensuring a mix of adult men, women and children. If fewer than five members were available, all willing were recruited. Sampling took place daily from 6 a.m. until 8 p.m. On certain days, sampling was scheduled to accommodate various routines, including the availability of individuals at work or school.

### Study instrument and data collection

Data were collected using a structured, interviewer-administered questionnaire collected using Research Electronic Data Capture (REDCap) on tablets [23,24]. Questionnaires were administered in either French or Malagasy and specifically developed and piloted for this study. At the household level, information was gathered on socioeconomic status, including access to electricity, primary cooking fuel (charcoal/wood, electricity, kerosene, or gas), and main lighting source (electricity, gas, kerosene, solar, or petrol). Water sources for drinking, bathing, laundry, and dishwashing, as well as type of toilet facility was gathered as proxies for schistosomiasis risk factors.

At the individual level, data included sociodemographic characteristics, occupation, risk behaviors, history of infection, and prior participation in MDA campaigns. To assess knowledge of schistosomiasis transmission, an additive knowledge score (range 0–12) was constructed from responses to 12 predefined statements: eight correct transmission routes (fishing, swimming, working in rice/agricultural fields, washing, collecting water, defecating or urinating in or near water, and poor sanitation contaminating water with stool or urine) and four commonly held misconceptions (drinking contaminated water, eating unwashed food, sexual contact, and sharing food or plates). Participants indicated whether each statement was true or false, earning one point per correct answer (correctly affirming true routes or rejecting false ones).

### POC-CCA testing

A urine sample (≥25 ml) was collected from all participants for Point of Care Circulating Cathodic Antigen (POC-CCA) testing. POC-CCA was performed on site according to the manufacturer’s (Rapid Medical Diagnostics) instructions [25]. Two drops (100 µl) of urine were transferred to the POC-CCA test cassette, and results were read after 20 min. Faint indication lines were deemed negative. Participants who tested positive were offered a one-time treatment of 40 mg/kg of PZQ following WHO recommendations [13].

### Data analysis

Continuous numeric data were described using the median and the interquartile range (IQR; shown as quartile 1– quartile 3). Categorical variables were described using frequencies and percentages. Due to missing values, the denominator may differ in some calculations.

A choropleth map, with the prevalence of positive test results by Fokontany was drawn. Getis and Ord’s G-statistics [26] were calculated to perform a cold/hot-spot analysis assessing the prevalence correlation among neighboring Fokontany, considering a set of four nearest neighbors. Knowledge of schistosomiasis transmission was assessed by calculating a score based on the number of correct responses regarding transmission risks. To assess the clustering of infections at household level, a bootstrap approach was applied. In each iteration, two individuals were randomly selected per household. The POC-CCA test result from the first individual was considered the outcome, and the test result from the second individual was treated as the exposure. This process was repeated 1000 times, and the prevalence ratio (PR) was calculated for each iteration. The median PR was reported as the point estimate, while the 2.5% and 97.5% percentiles of the PR distribution were used to define the 95% CI.

PRs were estimated to examine associations between schistosome infection positivity and individual risk factors. To address potential confounders, a hierarchical Poisson regression with robust variance [27] was conducted. The individual place of residence at Fokontany-level served as the second-level cluster variable. Due to convergence issues, the household level could not be considered as an additional cluster variable. The models’ goodness of fit was compared based on their Akaike information criterion (AIC). If the difference in AIC between two models was more than 2, the model with the smallest AIC score was considered. Random intercept models were ultimately employed. Interaction terms were considered in the regression model to assess the combined effect of two factors on the outcome. These variables were incorporated into both the crude and adjusted analysis, and no additional variable section procedures were applied. Only individuals with complete data on the regression variables were included in the risk factor analysis.

Hierarchical Poisson regression was performed using STATA 18 [28]. All other analyses were conducted in R using the *sp* and *osmdata* packages to manage spatial data and create maps, *spdep* to calculate G-statistics, and *epiR* to calculate PRs and estimate the sample size [29–32]. Boundary shapefile data to generate maps were taken from the Humanitarian Data Exchange (HDX) repository, and further geographic information was taken from OpenStreetMap [33,34].

## Results

### Description of the study sample

In total, 1,215 households from 42 Fokontany were included in the study. Table 1 shows the characteristics of the study households. A median of 25 (IQR = 17–37) households per Fokontany were sampled. Almost all households reported cooking with charcoal/wood (*n* = 1,197, 99%). The availability of electricity in households was low at 18% (*n* = 218). More than half of the households (*n* = 703, 58%) reported practicing personal hygiene outside their household in natural freshwater bodies, and only 38% (*n* = 463) reported having a toilet in their houses. Household characteristics were comparable within the Fokontany. For example, out of 42 Fokontany, 30 Fokontany had less than 10% of households with electricity, while 3 had more than 90% with electricity. Similarly, 21 Fokontany had less than 10% of household with a toilet, while 10 Fokontany had more than 90% with a toilet in their homes. Hence, living conditions depended on the respective Fokontany.

**Table 1.** Characteristics of the households included in the study.

Characteristics	Summary
Number of households	1,215
Number of Fokontany	42
Households per Fokontany [median (IQR, range)]	25 (17–37, 8–84)
Individuals per household [n/N (%)] 1 2 3 4 5	106/1,215 (9) 40/1,215 (3) 94/1,215 (8) 143/1,215 (12) 832/1,215 (68)
Cooking with charcoal/wood [n/N (%)]	1,197/1,215 (99)
Having electricity [n/N (%)]	218/1,210 (18)
Solar lamp as the main light source [n/N (%)]	762/1,214 (63)
Well (with pump) as main water source [n/N (%)]	560/1,214 (46)
Washing laundry in natural water bodies [n/N (%)]	791/1,214 (65)
Showering/bathing outside [n/N (%)]	703/1,213 (58)
Toilet at home [n/N (%)]	463/1,210 (38)

From these households, 5,200 individuals were recruited, resulting in a median of 5 (IQR = 4–5) individuals per household (Table 2). Slightly more females participated (*n* = 2,926, 56%). The median age was 27 years (IQR = 15–55). Half of participants reported to have completed primary education (*n* = 2,739, 53%), and 71% (*n* = 3,673) reported being employed at the time of the survey. The POC-CCA test was positive for 59% (*n* = 3,042) of participants. Despite the high number of POC-CCA positives, only a small fraction of participants had been diagnosed and treated for schistosomiasis in the past year (*n* = 23, <1%) or more than one year ago (*n* = 72, 1%).

**Table 2.** Characteristics of the individuals included in the study.

Characteristics	Summary
Number of individuals	5,200
Female sex [n/N (%)]	2,926/5,198 (56)
Age in years [median (IQR, range)]	27 (15–44, 5–99)
Never been to school	432/5,175 (8)
Primary education	2,739/5,175 (53)
Secondary education	1,836/5,175 (35)
University degree	168/5,175 (3)
Reported occupation [n/N (%)]	3,674/5,200 (71)
Reported occupation, if >5% [n/N (%)]:	
Farmer	1,733/3,674 (47)
Housewife/househusband	384/3,674 (10)
University student	296/3,674 (8)
Seller	288/3,674 (8)
Craftsman	188/3,674 (5)
*S*. detected and treated with Praziquantel within the last year [n/N (%)]	23/5,200 (<1)
*S*. detected and treated with Praziquantel more than one year ago [n/N (%)]	72/5,200 (1)

Fig. S1A provides details about the study participants’ occupation. The majority (*n* = 1,733/3,674; 47%) reported working in agriculture. Multiple responses were possible to define the agricultural sector in which participants reported to be working in (Fig. S1B). Most farmers reported working in rice cultivation (*n* = 1,711/1,733, 99%). Agricultural sectors other than rice farming were reported to a much lesser extent. However, the majority of farmers who reported working in other agricultural sectors reported working in rice farming as well. Figure S1C shows that 100% of zebu and pig farmers, and about 90% of the farmers working with other animals also reported working in rice farming. Most rice farmers reported working in their own rice fields (n/N = 1,649/1,711, 93%) of which 21% (n/N = 345/1649) reported working in fields owned by other farmers as well. Among rice farmers, 38% (619/1,624) reported having 3 to 6 hours of freshwater contact during farming, while 60% (988/1,624) reported more than 6 hours of freshwater contact per day due to farming activities. Figure S2 summarizes schistosomiasis prevalence by reported occupation and age group.

### Geospatial analysis of schistosome distribution and MDA programs

The prevalence of schistosome infection was estimated at Fokontany level. The median prevalence across all Fokontany was 61% (IQR = 40–83). POC-CCA test positivity strongly varied across Fokontany (Figure 1(B)). A hot/cold-spot analysis was performed using G-statistics to find geospatial clustering of prevalence values (Figure 1(C)). The Fokontany belonging to a hot- or cold-spot cluster with a p-value <0.1 is marked with a bold outline. The map indicates a subtle north-south trend, with a higher likelihood of lower prevalence estimated for neighboring Fokontany in the north-central study area, while a higher likelihood of higher prevalence was observed in the southern study region.

### Analysis of factors influencing schistosome infection

**Figure 2.**
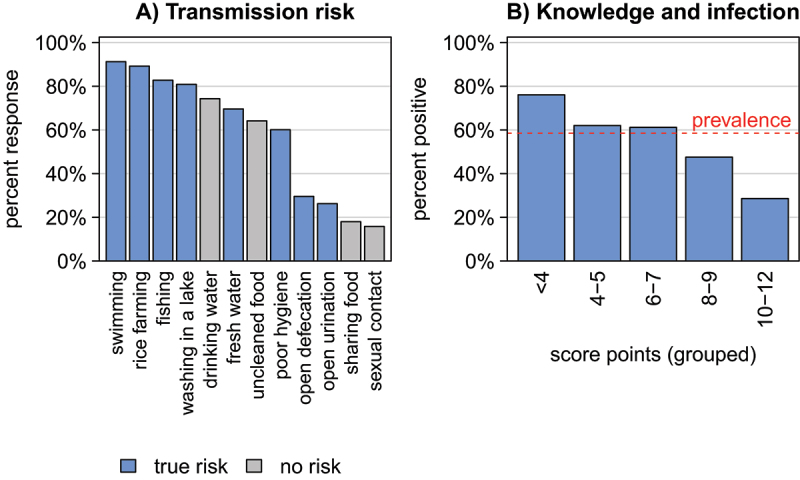
Individual transmission knowledge and schistosomiasis risk: (A) knowledge of schistosomiasis transmission and (B) schistosomiasis prevalence among participants grouped by knowledge score (overall prevalence is depicted by the dashed line).

Knowledge and socio-demographic factors were analyzed to identify specific factors associated with a higher risk of schistosome infection. Knowledge of schistosomiasis transmission is shown in Figure 2(A). Most participants correctly named swimming in freshwater bodies (n/N = 4,720/5,190, 91%), rice farming (n/N = 4,620/5,178, 89%), fishing (n/N = 4,298/5,193, 83%), and washing (e.g. laundry or dishes) in a freshwater river or lake as risk factors (n/N = 4,192/5,185, 81%). In contrast, less frequently mentioned incorrect responses included food sharing (18%, n/N = 932/5,186) and sexual contact (16%, n/N = 818/5,180). Based on these responses, a simple additive knowledge score was generated. Figure 2(B) illustrates the risk of infection by knowledge score. The highest prevalence of schistosome infection was observed in the group with a score of <4 points (76%, n/N = 200/263), while the lowest prevalence was found in the group with the highest knowledge (≥10 points) (29%, n/N = 74/259).

The proportion of positive POC-CCA tests varied by reported occupation, ranging from 74% (*n* = 1,276/1,732) in farmers to 41% (n/N = 121/296) in university students, indicating that participants mainly working in agriculture had the highest risk of infection (Fig. S2A). The proportion of positive POC-CCA tests was similar among rice farmers who reported 3–6 h of freshwater contact during their work and those who reported more than 6 h per day (PR = 0.97, 95%-CI: 0.91–1.03). Schistosome infection prevalence showed a positive trend across age groups (Fig. S2B), from 44% (n/N = 593/1,333) in children under 15 years to 70% (n/N = 540/773) in participants aged 45–60 years.

To evaluate the clustering of schistosome infection at the household level, the risk of a positive test result depending on the test outcome of other household members was estimated. The likelihood of being tested positive increased by 76% (95% CI: 46%–129%) when another household member was also tested positive, suggesting clustering of infection within households.

**Figure 3.**
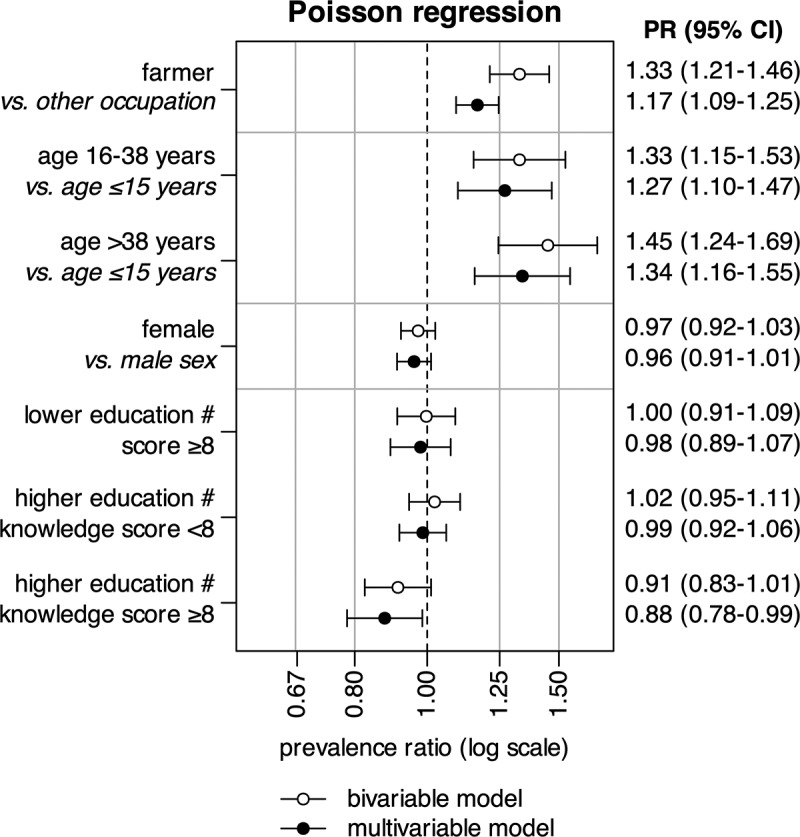
Bi- and multivariable models of associations with a positive POC-CCA test, with interaction terms denoted by a #. A numeric summary is provided in supplementary table S1.

Individual risk factors associated with positive POC-CCA test results were assessed using hierarchical Poisson regression (Figure 3 and Table S3). Of the study participants, 5,155 (99%) had a complete set of data on the regression variables and were included in the regression analysis. Adjusted PRs show a moderate risk increase in farmers (PR = 1.17, 95% CI: 1.09–1.25) and older age groups (16–38 years, PR = 1.27, 95% CI: 1.10–1.47 and ≥38 years, PR = 1.34, 95% CI: 1.16–1.55; both versus ≤15 years). Education or knowledge of transmission had no effect on POC-CCA test results; however, in the interaction analysis the combined effect of higher formal education and higher knowledge score (i.e. higher education # knowledge score ≥8) showed a slightly reduced risk of infection (PR = 0.88, 95% CI: 0.78–0.99). The results of the full regression model are comparable with those of the crude analysis.

## Discussion

This cross-sectional study reports a high prevalence (59%) of schistosome infections in a region of Madagascar where ≥80% MDA coverage was reported in 2021 among SAC [16]. Knowledge of schistosomiasis and its transmission is relatively high. Combined higher education and greater knowledge are associated with a reduced risk of infection. Our data show that to reach the WHO goal of eliminating schistosomiasis as a public health problem by 2030, interventions in Madagascar and potentially in other hyperendemic regions with similar characteristics need to be revised and adapted to specific contexts [9].

According to WHO guidelines for the control and elimination of human schistosomiasis, in areas with a prevalence above 10%, all individuals at risk, regardless of age, should be targeted by MDA [13]. However, if the observed prevalence does not decrease effectively, MDA should be upgraded to biannual administration, as in the case for the District of Vatomandry, though this has not yet been implemented [13,16].

Despite this recommendation, the present study found no strong risk factors for schistosome infection, except for working in agriculture. Farmers had an 18% increased risk of schistosome infection compared to other participants. Older age groups were also more likely to be infected. In rural Madagascar, as in many schistosomiasis-endemic countries [35–37], farming is the main occupation, contributing to an overall high risk of infection [17,38]. Consequently, adopting a risk-based approach for MDA, which would translate into treating the entire population, may not be realistic due to the financial effort required [39] and the limited availability of PZQ [40]. This underscores the urgent need for alternative approaches, such as environmental interventions or the modification of risk behaviors, as recommended in the WHO schistosomiasis guidelines (Recommendations 4 and 5) [13].

By mapping the geospatial distribution of infection, we could identify prevalence variations that may reflect community and infrastructural factors influencing the spread and transmission of the disease. However, schistosome infection did not follow a clear geographical pattern, as the high-prevalence areas identified are scattered throughout the entire district. An assessment of existing infrastructure at Fokontany level, including access to electricity and sanitation facilities, revealed no strong differences between households, as most reported poor living conditions. This finding aligns with current literature, which describes poor sanitation and living conditions as one of the primary factors perpetuating schistosomiasis [1,41].

The World Health Assembly (WHA) resolution 54.19 from 2002 states that MDA programs to combat schistosomiasis sufficiently must be paired with WASH interventions to reduce reinfection risk, as MDA alone is insufficient [42]. Several studies support the effectiveness of comprehensive interventions [43,44], but implementing WASH measures faces several challenges, including poor support from political leaders and low community compliance [41,45]. Our data reveal that, despite high prevalence of infection, there exists fair knowledge about schistosomiasis transmission in the study area. However, only those with higher formal education show reduced infection risk, suggesting that health education should be tailored to different educational levels ensuring that participants from different educational backgrounds are able to translate information into effective health behavior. Interestingly, we also see a relatively low awareness of MDA occurring in the region (Figure 1(D)), despite exposure to treatment. As observed in Madagascar [46], health education efforts should be revised to be more context-specific in order to enhance the adoption of preventive behaviors among populations at risk.

Our findings also confirm that schistosomiasis can be classified as an occupational disease, with rice farming contributing significantly to the transmission and incidence of infection (Table S3) [47]. According to our data, protective measures such as wearing gloves or boots are not widely adopted by farmers, even though transmission patterns are known.

Previous studies have shown that efforts to enforce the use of such equipment among farmers in endemic countries have largely failed [48–50]. Schistosomiasis is primarily a chronic disease in endemic areas, so its acute symptoms and immediate consequences are not instantly tangible to those at risk and those who are infected, reducing the motivation for adopting prevention measures [51–53]. Given the tropical climate and harsh working conditions in most endemic countries, workers are often unwilling to protect themselves with uncomfortable equipment that needs to be maintained, even if they are aware of the presence of the disease. Recent studies have clearly shown that awareness alone is not enough to promote behavioral changes that are crucial for effective and sustainable disease prevention [54–57]. It is becoming increasingly clear that ownership of intervention measures and the immediate perception of a direct benefit are crucial for the promotion of preventive healthcare [58,59]. In this view, alternative strategies must be explored and tested in order to promote those behavioral changes that seem critical to effectively combat schistosomiasis.

Our study shows a positive association between schistosome infection and age (Figure 3). Infections in individuals beyond school age are more likely to go untreated and progress to chronic, complicated forms of the disease. Prolonged exposure can lead to conditions like liver fibrosis (from *S. mansoni*) or female genital schistosomiasis (from *S. haematobium*), which are difficult to diagnose and treat, particularly in endemic areas [17,51,53]. These conditions also contribute to a general loss of work productivity, feeding the vicious cycle of poverty that is typical of schistosomiasis and other NTDs [1].

The highly standardized study procedures, continuous staff training, quality assurance measures, and large sample size allowed precise inferences about the prevalence of schistosome infection. However, our study does not come without limitations. First, while the study was designed to assess individual-level infection risk, living conditions and behaviors within Fokontany showed little variation, making it difficult to pinpoint specific risk factors. Second, schistosome infection was detected using POC-CCA test. Despite the parasitological methods recommended by WHO guidelines [13], its application for MDA programs’ purposes remains controversial due to limitations in interpreting results, particularly for low-intensity infections [60], its limited use for the detection of *S. mansoni* and lack of robust data to translate findings from the test into recommendations for public health interventions [61]. Furthermore, excluding inaccessible Fokontany to guarantee security of the staff may have created a blind spot overlooking more remote areas with higher prevalence, lower MDA coverage, and poorer socioeconomic conditions. Finally, due to the cross-sectional nature of the study, we are unable to conduct a detailed analysis of exposure histories to explain the reasons behind associations such as age trends. Past exposures that are not captured by the study design could be the cause of chronic infections in older individuals.

In conclusion, our study demonstrates that, in order to meet the WHO target of eliminating schistosomiasis as a public health problem by 2030, adapted approaches toward MDA distribution need to be implemented and combined with innovative and tailored interventions to prevent and control the disease. The high prevalence in the District of Vatomandry in all age groups, combined with the lack of clear risk factors for infection, suggests that in such contexts, MDAs alone might not suffice to eliminate the disease as a public health problem. Furthermore, empowering communities and fostering active participation, in shaping the implementation of interventions aimed at preventing schistosome infection while simultaneously enhancing productivity can serve as the cornerstones for achieving the elimination goal by 2030.

## Supplementary Material

Figure S1.pdf

Figure S2.pdf

Table_S3.docx
