# Supplementary material for: Prevalence of schistosome infection in a region of Madagascar regularly undergoing mass drug administration: a cross-sectional study
Source: Pathog Glob Health. 2026 Feb 2;120(2):130–9. doi: 10.1080/20477724.2026.2616620 (PMC13137748; doi:10.1080/20477724.2026.2616620)
Supplement: Figure S1.pdf [file YPGH_A_2616620_SM3188.pdf]

Figure S1: Occupation of the of the study participants and details on farming activities

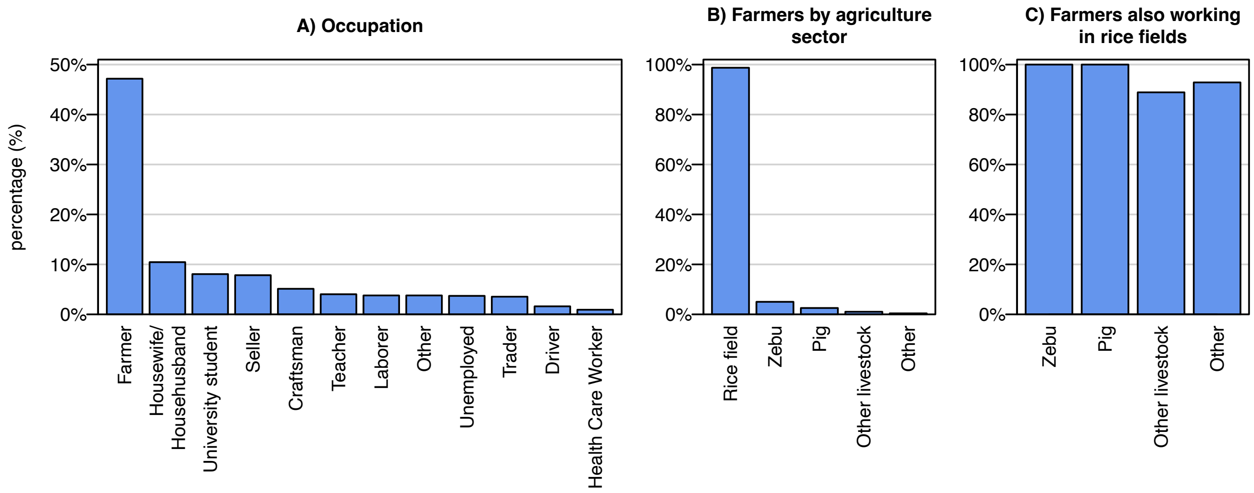

Legend: A) shows the percentage of study participants working in different occupations, B) shows the agriculture sector within the farmers work (multiple responses possible), and C) shows the proportion of non-rice farmers, who also work in rice farming at times.

Occupation category "other" (n>10) includes fisher, 37 (27%); chief of Fokontany/mayor, 23 (17%); security guard, 19 (14%); retired, 13 (9%), and household help, 11 (8%).
