# Supplementary material for: Prevalence of schistosome infection in a region of Madagascar regularly undergoing mass drug administration: a cross-sectional study
Source: Pathog Glob Health. 2026 Feb 2;120(2):130–9. doi: 10.1080/20477724.2026.2616620 (PMC13137748; doi:10.1080/20477724.2026.2616620)
Supplement: Figure S2.pdf [file YPGH_A_2616620_SM3187.pdf]

Figure S2: Schistosomiasis prevalence by reported occupation and age group.

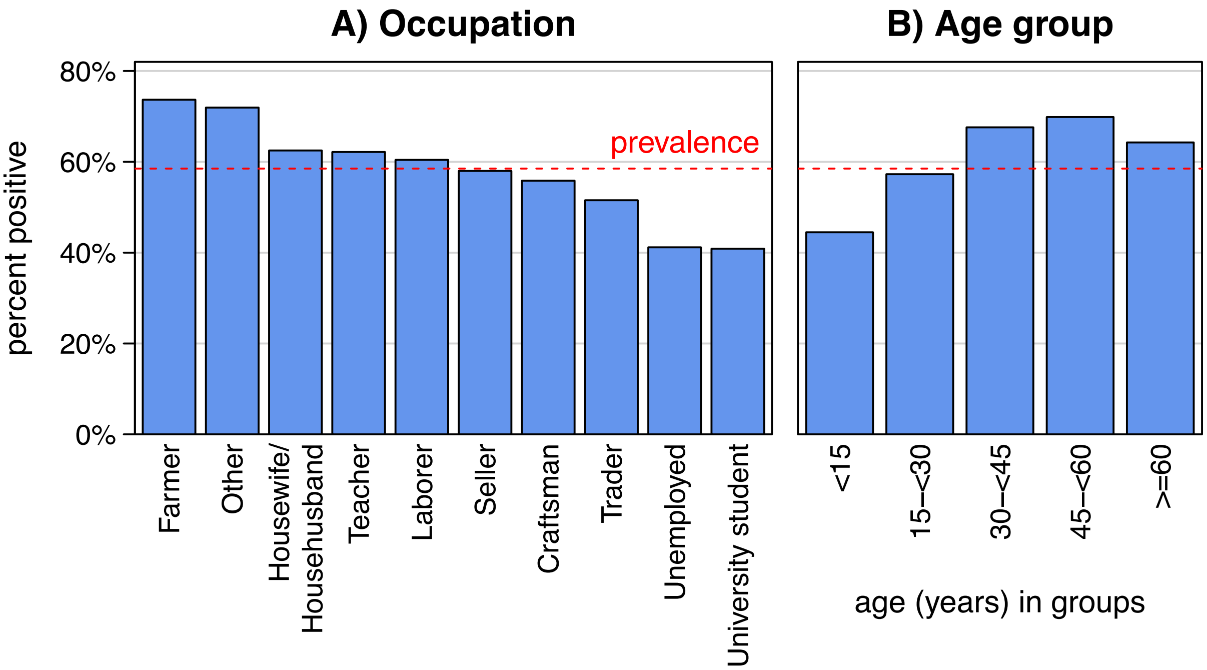

Legend: A) shows the schistosomiasis prevalence by reported occupation and B) shows the schistosomiasis prevalence by reported age group. The overall prevalence estimated by POC-CCA is depicted by the dashed line in red.
