# Supplementary material for: Prevalence of schistosome infection in a region of Madagascar regularly undergoing mass drug administration: a cross-sectional study
Source: Pathog Glob Health. 2026 Feb 2;120(2):130–9. doi: 10.1080/20477724.2026.2616620 (PMC13137748; doi:10.1080/20477724.2026.2616620)
Supplement: Table_S3.docx [file YPGH_A_2616620_SM3186.docx]

**Table S3:** Crude and adjusted hierarchical Poisson regression models (clustering on Fokontany level) on the risk for a positive POC-CCA test. This is the numeric representation of the results shown in Figure 3.

|  | **positive**  (n=3,014, 58%) | **negative**  (n=2,141, 42%) | **Crude PR** | **Adjusted PR** |
| --- | --- | --- | --- | --- |
| **farmer** | 1252 (73%) | 453 (27%) | 1.32 (1.22-1.44) | 1.18 (1.09-1.27) |
| ***other occupation*** | 1762 (51%) | 1688 (49%) | ref. | ref. |
|  |  |  |  |  |
| ***age ≤15 years*** | 592 (45%) | 738 (55%) | ref. | ref. |
| **age 16–38 years** | 1,322 (60%) | 886 (40%) | 1.33 (1.16-1.52) | 1.28 (1.11-1.47) |
| **age ≥38 years** | 1,100 (68%) | 517 (32%) | 1.46 (1.26-1.69) | 1.36 (1.17-1.56) |
|  |  |  |  |  |
| **female sex** | 1,708 (59%) | 1,192 (41%) | 0.98 (0.92-1.03) | 0.96 (0.91-1.02) |
| ***male sex*** | 1,306 (58%) | 949 (42%) | ref. | ref. |
|  |  |  |  |  |
| ***Lower education # score<8*** | 1,130 (67%) | 544 (33%) | ref. | ref. |
| **Lower education # Score≥8** | 818 (55%) | 666 (45%) | 0.95 (0.85-1.07) | 0.94 (0.84-1.05) |
| **Higher education # score<8** | 505 (63%) | 298 (37%) | 1.02 (0.94-1.10) | 0.98 (0.91-1.06) |
| **Higher education # score ≥8** | 561 (47%) | 633 (53%) | 0.88 (0.78-1.00) | 0.85 (0.74-0.97) |
